# Baron Larrey on Actual Cautery in Traumatic Erysipelas

**Published:** 1826-07

**Authors:** 


ACTUAL CAUTERY IN TRAUMATIC ERYSIPELAS. BY BARON LARREY.

The Baron observes that, on the fourth or fifth day after the infliction of a wound, there must necessarily arise an inflammation on the borders and parietes of the said wound which produces a too speedy union, especially if the division of continuity be not simple and uniform, like that made by the surgeon's instruments. Improper applications, the contact of cold air, bilious irritation, and many other causes, increase this inflammation, which often becomes of an erysipelatous character, and spreads rapidly to other parts. If there be any deleterious miasmata in the atmosphere at the time, the mischief is increased, and the erysipelas often ends in gangrene. If the biliary secretion be in a bad state, the erysipelas assumes an unhealthy aspect—the wound becomes yellow at the bottom, and hepatitis is often a complication. If the stomach be in a state of debility, the erysipelas becomes pale, the edges of the wound tumid, and the base covered with a thick yellowish and putrescent substance, characteristic of debility and hospital gangrene. To this species of erysipelas Baron Larrey applies the actual cautery, near to the wound, and affirms that it arrests at once the progress of the phlegmasia. The application causes very little pain, and is accompanied and succeeded—1st, by a gaziform effluvia of an animal odour—2d, by the disappearance of the heat and tensive pain of the part inflamed—3d, by the dissipation also of the redness and swelling of the part,—4thly, these cauterisations are not followed by any suppuration, or gangrene, the burnt skin merely peeling off in carbonaceous crusts, leaving no sensible cicatrices; 5thly, the purulent discharge from the wound, which had ceased on the appearance of the erysipelas, is soon reproduced; 6th, the strength of the patient is increased, the functions of the viscera re-established, and the exanthematic disease put a stop to. The hot iron is to be applied to the reddest points of the erysipelas. Some cases in illustration are related by the Baron, but they need not be noticed in this place.—*Révue Medicale, Fevrier*, 1826.

